# Study Protocol for a Randomized, Double-Blind, Community-Based Efficacy Trial of Various Doses of Zinc in Micronutrient Powders or Tablets in Young Bangladeshi Children

**DOI:** 10.3390/nu10020132

**Published:** 2018-01-26

**Authors:** M. Munirul Islam, Christine M. McDonald, Nancy F. Krebs, Jamie Westcott, Ahmed Ehsanur Rahman, Shams El Arifeen, Tahmeed Ahmed, Janet C. King, Robert E. Black

**Affiliations:** 1 Nutrition and Clinical Services Division, International Centre for Diarrhoeal Disease Research, Bangladesh (icddr,b), Dhaka 1212, Bangladesh; tahmeed@icddrb.org; 2 Children’s Hospital Oakland Research Institute, Oakland, CA 94609, USA; chmcdonald@chori.org (C.M.M.); jking@chori.org (J.C.K.); 3 Department of Pediatrics, University of Colorado Denver Anschutz Medical Campus, Aurora, CO 80045, USA; Nancy.Krebs@ucdenver.edu (N.F.K.); Jamie.Westcott@ucdenver.edu (J.W.); 4 Maternal and Child Health Division, International Centre for Diarrhoeal Disease Research, Bangladesh (icddr,b), Dhaka 1212, Bangladesh; ehsanur@icddrb.org (A.E.R.); shams@icddrb.org (S.E.A.); 5 Institute for International Programs, Bloomberg School of Public Health, Johns Hopkins University, Baltimore, MD 21205, USA; rblack1@jhu.edu

**Keywords:** zinc, multiple micronutrient powder, diarrhea, linear growth, infants, exchangeable zinc pool size

## Abstract

Zinc is essential to supporting growth in young children especially for tissues undergoing rapid cellular differentiation and turnover, such as those in the immune system and gastrointestinal tract. Therapeutic zinc supplementation has been initiated in low-income countries as part of diarrhea treatment programs to support these needs for young children, but the effects of preventive supplemental zinc as a tablet or as a multiple micronutrient powder (MNP) on child growth and diarrheal disease are mixed and pose programmatic uncertainties. Thus, a randomized, double-blind community-based efficacy trial of five different doses, forms, and frequencies of preventive zinc supplementation vs. a placebo was designed for a study in children aged 9–11 months in an urban community in Dhaka, Bangladesh. The primary outcomes of this 24-week study are incidence of diarrheal disease and linear growth. Study workers will conduct in-home morbidity checks twice weekly; anthropometry will be measured at baseline, 12 weeks and 24 weeks. Serum zinc and other related biomarkers will be measured in a subsample along with an estimate of the exchangeable zinc pool size using stable isotope techniques in a subgroup. Therapeutic zinc will be provided as part of diarrhea treatment, in accordance with Bangladesh’s national policy. Therefore, the proposed study will determine the additional benefit of a preventive zinc supplementation intervention. The protocol has been approved by the Institutional Review Boards (IRBs) of icddr,b and Children’s Hospital Oakland Research Institute (CHORI). The IRB review process is underway at the University of Colorado Denver as well.

## 1. Introduction

Zinc is an essential mineral in humans [1]. Zinc deficiency is common in low- and middle-income countries (LMICs), thought to be primarily due to an inadequate zinc intake [2]. A large number of randomized controlled trials of daily supplementation with zinc in the form of dispersible tablets or syrups have been conducted in children in LMIC; meta-analyses of these trials have demonstrated a significant reduction in diarrhea and improved linear growth [3,4]. Some trials have also reported reductions in pneumonia, hospitalizations, and deaths [3,5,6].

Zinc is a component of multiple micronutrient powders (MNPs), which are added to complementary foods consumed by older infants and young children [7]. MNPs are intended to improve vitamin and mineral intakes and address key micronutrient gaps including zinc and iron [8]. MNPs currently contain 5 mg or 4.1 mg of zinc, in the standard 5-component or 15-component MNP, respectively [7].

MNP programs are rapidly being scaled up globally for delivering iron to older infants and young children [9]. However, concerns about potential adverse outcomes with MNPs have been expressed. For example, MNP supplementation of Pakistani children under 2 years of age increased in the number of days with diarrhea compared to controls even in the group consuming an MNP with 10 mg of zinc [10]. This finding was supported by a systematic review of 17 studies, which showed that MNPs significantly increased the incidence of diarrhea [11]. The deleterious effects of MNPs may be due to iron, as several studies have reported that iron supplementation increased the incidence of diarrhea [10,12,13,14]. Research has also shown that the administration of MNPs to children under 2 years of age in LMICs had no effect on anthropometric indices [15]. The effect of MNPs containing zinc and iron on serum zinc concentrations is inconclusive [10,12,13,14]. The bioavailability of these trace elements from MNPs may be impaired by components such as phytate in the foods to which the MNPs are added. However, a recent study showed that giving iron and zinc together in MNPs did not reduce zinc absorption [16].

The objectives of this research are to determine the optimal physical form (i.e., powder or dispersible tablet), dose, and frequency of preventive zinc supplementation and the best way to co-administer zinc with iron for reducing diarrhea and improving growth in older infants and young children. To achieve these objectives, we propose to compare the following groups of children in a randomized, partially double-blind, controlled, community-based efficacy trial in a low-income urban population of Dhaka, Bangladesh: (1) standard 15-component MNPs consumed daily; (2) high-zinc, low-iron MNPs containing the same micronutrients as study group 1, except with 10 mg instead of 4.1 mg of zinc and 6 mg instead of 10 mg of iron, consumed daily; (3) high-zinc (10 mg instead of 4.1 mg) MNPs with low iron (6 mg instead of 10 mg) and high-zinc, iron-free MNPs consumed on alternating days; (4) dispersible tablet with 10 mg zinc only, consumed daily; (5) dispersible tablet with 10 mg zinc only, consumed daily for two weeks at the beginning and at the 3-month point of the trial and placebo tablets on all other days; and (6) placebo MNP consumed daily. Rationales for selection of the aforementioned intervention groups have been detailed in Section 2.3. Additionally, we also will measure the exchangeable zinc pool (EZP) size in response to supplementation in a sub-group of children from the study population who will be randomly allocated to groups 2, 4 and 6, and to measure serum zinc, serum ferritin, serum transferrin receptor (sTfR), serum retinol binding protein (RBP), serum alpha 1-acid glycoprotein (AGP), serum c-reactive protein (CRP) and hemoglobin in all six groups.

Incidence of diarrhea and change in length-for-age *z*-score (LAZ) are the primary outcomes of the trial. EZP size, a measure of zinc nutrition, and biomarkers of zinc and iron status will be secondary outcomes to be evaluated in subgroups of study participants. Since therapeutic zinc supplementation is recommended by the World Health Organization (WHO) and Bangladesh’s national policy as part of treatment for diarrhea, all children will receive therapeutic zinc supplementation along with Oral Rehydration Solution (ORS) for treatment of diarrhea as part of the study protocol [17]. As such, the proposed study will examine the incremental benefit of preventive zinc supplementation on the study outcomes over and above what is currently afforded by therapeutic zinc supplementation for treating diarrhea.

## 2. Methods

### 2.1. Study Setting

The study will be conducted in the peri-urban, low-income area of Mirpur in Dhaka, Bangladesh. This area was selected for the study given that it is only ~10 km from the icddr,b, and the prevalences of zinc deficiency (serum zinc < 9.9 mmol/L), diarrhea and stunting among preschool-aged children in the urban slums of Dhaka reported in the National Micronutrient Survey 2011–2012, were 51.7%, 10% and 51.1%, respectively [18]. The population of Mirpur is approximately 500,000; therefore, it will be possible to enroll the desired number of participants from this study site within 12 months. This area in Dhaka is served by a Community Health Centre/Research Field Office (CHC/RFO) operated by the icddr,b and several other scientific research projects are currently being conducted there as well. The study will recruit a total of 2886 children as study participants.

### 2.2. Study Participants

Children will be eligible to participate in the trial if they are 9–11 months of age at the time of enrolment and have a weight-for-length *z*-score (WLZ) ≥ −3 according to the 2006 WHO Growth Standards [19]. Children will be excluded from the trial if they have any of the following conditions: (1) Severe Acute Malnutrition, defined as WLZ < −3 and/or the presence of bipedal edema and/or mid-upper arm circumference (MUAC) < 115 mm; (2) Congenital anomalies (e.g., cardiac defects, cleft lip or palate) or any other conditions that interfere with feeding; and (3) Chromosomal anomalies and other organic problems (e.g., jaundice, tuberculosis, etc.). The 9–11 month age range was chosen for study because the beneficial impact of zinc supplementation on growth and on the incidence of diarrhea appears to be greatest during the second year of life (i.e., 12–24 months). Furthermore, maximal growth responses are usually seen among children less than 2 years of age. In addition, children under 2 years of age are a high-risk group for zinc deficiency in Bangladesh and other South Asian countries, given their usual dietary practices primarily of plant-origin [20].

### 2.3. Study Interventions

The study will have six intervention groups with varying doses, forms and/or frequencies of zinc supplementation; the duration of the intervention is 24 weeks for all arms. Table 1 lists the details of the supplement in each study group. All powder formulations will be manufactured by the DSM India Private Limited (Haryana, India). The dispersible zinc and placebo tablets will be manufactured by Nutriset (Malaunay, France). Study group 1 will consume the standard, 15-component MNP, which is currently recommended for areas where the prevalence of anemia is ≥20% [21]. Because this standard MNP formulation with 4.1 mg zinc has not been shown to affect linear growth, as occurs with higher zinc supplementation doses, and because MNPs containing 10 mg of iron have been associated with an increase in the incidence of diarrhea [15], study group 2 will consume a high-zinc, low-iron MNP formulation to determine if a MNP formulation with a lower iron: zinc ratio (3:5 in group 2 versus 10:4.1 in group 1) improves growth while reducing diarrhea. Participants assigned to study group 3 will consume the high-zinc, low-iron MNP formulation and a high-zinc, no-iron MNP formulation on alternating days (i.e., on day 1 consumes high-zinc, low iron MNP formulation then on day 2 will consume high zinc, no iron MNP formulation, then on day 3 again the same formulation as on day 1 and so on) to determine the impact of giving iron and zinc on alternate days on growth and diarrhea prevention. Since several studies have documented the positive impact of preventive zinc supplementation in the form of a dispersible tablet [3,22], study group 4 will receive a 10 mg dispersible zinc tablet, which will serve as the positive control. Study group 5 will assess whether intermittent consumption of 10 mg of preventive zinc supplements for 14 days immediately following enrolment, for 14 days at 3 months and placebo tablets on all other days’ results in sustained benefits on diarrhea prevention and growth. Finally, study group 6 will receive a placebo powder.

### 2.4. Screening and Enrollment

Trained study workers will survey the communities and the surrounding households close to the study site to identify children who are between 9 and 11 months of age and to screen for the exclusion criteria mentioned above. Surveys will be repeated during the study period as required until enough participants are identified to meet the specified sample size. Children will be screened for exclusion criteria, and then the parents of eligible children will be invited to bring their child to the Research Field Office (RFO) so that a study physician can verify eligibility and consent can be obtained. If more than one eligible child is present in each household, both children will be enrolled in the same trial group; however, one of the two children will be randomly designated as the “study child”, and only his/her data will be entered into the database.

In a dedicated room in the RFO, a study physician will explain the study objectives to the caregivers in detail and perform a thorough physical examination of the potential study participants to assess their health status including presence of any congenital anomalies and acute or chronic illness(s), as mentioned above. The child’s age will be verified against documentation (birth certificate or immunization card, if available) or the caregiver’s report of the child’s birth date; it will also be cross-checked with religious or major social events, if necessary. A trained study anthropometrist assisting the study physician will measure the child’s length and weight using standard methods described below. LAZ and WLZ will be calculated following the 2006 WHO Growth Standards [17]. After the physician has confirmed the child’s eligibility, written informed consent will be sought from one or both parents or the primary caregiver by the study staff.

### 2.5. Consent Procedures

The consent process will be in Bangla, the national language of Bangladesh that is universally spoken and understood in Bangladesh. The Field Research Supervisors will be primarily responsible for the informed consent process, and in all cases will confirm consent and respond to any questions from participants prior to completion of the process. The initial components of the consent process overlap with detailed eligibility assessment, and thus will be overseen by the physician. However, other trained study workers (i.e., Field Research Assistants (FRAs)) may assist in providing detailed explanations of study procedures, risks, and benefits to the prospective participants.

Literate women or their family members will be encouraged to read the consent form aloud, under the supervision of study personnel. Alternatively, because of variable levels of literacy, consent documents will be read to prospective participants by study personnel if necessary. If a woman is interested in enrolling her child in the study, she will be given a consent form and asked to review it with her husband and/or family members. Prospective participants may take several days to consider participation, if they would like to. A copy of the model consent form is provided in Appendix A and Appendix B.

If a participant’s caregiver withdraws consent at any point of the study, all study procedures will be stopped. Data on participants/caregivers who refuse to continue in the study will be included in the analysis on an intention-to-treat basis. However, the primary outcome data for such participants, if missing, will not be imputed.

### 2.6. Follow-Up Procedures

Trained FRAs will visit the study child and his/her caregiver at their home twice per week throughout the 24-week study period. At one of these twice-weekly visits, the FRA will provide the child’s caregiver with the prescribed study supplement for seven days. If the child is in study groups 1, 2, 3, or 6, the FRA will instruct the mothers to mix the daily dose of MNP or placebo powder into the first few portions (preferably 1–2 tablespoons) of the child’s food in a single meal each day of the week. If the child is in study groups 4 or 5, respective mothers will be instructed to place the dispersible tablets in approximately one half tablespoon (5–10 mL) of expressed breast milk or clean water, wait until the tablet dissolves, and then feed the entire amount of the liquid in one spoonful to the child. The mothers will be instructed to follow this process daily for the 24-week study.

If a child enrolled in the trial develops diarrhea, he/she will receive standard treatment, including a 20 mg therapeutic zinc supplementation in the form of a dispersible tablet for 10 days. The mother will be instructed how to give the 20 mg zinc supplement in one of two ways. If the study worker diagnoses diarrheal disease during one of the household visits, (1) the study worker will provide therapeutic zinc and ORS to the child’s caregiver and instruct the caregiver to withhold the usual study regimen until the diarrheal treatment course has been completed (which is typically 10 days); (2) if a child experiences diarrhea (or any other illness) between the semi-weekly household visits, the child’s caregiver will be instructed to bring the child to the day clinic at the Field Research Office, which will be staffed by a study physician. This physician will provide the child with therapeutic zinc and ORS for diarrhea treatment and will instruct the caregiver to withhold the usual study regimen. The physician will inform the study worker who normally visits the child’s home and the corresponding supervisor, so the child’s treatment can be monitored and the study regimen can be resumed upon completion of treatment.

The zinc and iron content of all study interventions and placebo products will be analyzed at three time points (i.e., at the beginning of the study, at the middle of the recruitment and at the end of the study), in order to document that the iron and zinc content of the supplements are consistent with the study protocol. At least 10 random samples will be analyzed at each time point. The results will not be provided to study investigators to maintain blinding unless any of the six study regimens contain ±5% of the desired amount of zinc. This laboratory analysis will be performed using flame atomic absorption spectrophotometry with a deuterium arc background correction lamp (AAS, Perkin-Elmer Corporation, model AAnalyst 400, Norwalk, CT, USA) at the Pediatric Nutrition Laboratory at the University of Colorado Denver.

### 2.7. Study Outcomes

The first primary outcome variable is the diarrheal incidence over the study period of 24 weeks. Diarrheal incidence will be expressed as the number of episodes (as defined above) per child during the 24-week study period. Secondary outcomes linked to diarrhea include the prevalence of diarrhea, incidence of dysentery, incidence of diarrhea with dehydration, and all hospitalizations. The other primary outcome variable will be change in linear growth defined by LAZ over the 24-week study period, i.e., the absolute change in LAZ from enrolment to the end of the 24-week study period.

### 2.8. Participant Recruitment Plans and Study Timeline

Study participants will be recruited and enrolled on a monthly basis. Given the sample size described below, our targeted enrolment rate is 241 children per month; we anticipate completing enrolment in 12 months. Each participant will be studied 24 weeks. The total timeline for the study will be approximately 27 months, excluding time for manuscript publication in peer-reviewed journals. A schematic of the proposed timeline is provided in Appendix B.

### 2.9. Sample Size Calculations

Diarrheal incidence over a 24-week period is expected to follow an overdispersed Poisson distribution; the mean incidence from 9 to 15 months is expected to be 2.1 episodes, and the overdispersion parameter is 1.56 (based on prior data from the same study area); the statistical test will involve comparing six means simultaneously at a 5% level of significance. In order to detect a 20% difference between the mean diarrheal incidence in any two groups with 80% power, a sample size of 418 participants per group will be needed. When this is inflated by 15% to account for possible attrition and sub-optimal adherence to the study interventions, the final sample size is 481 participants per group (i.e., 2886 participants total).

The sample size of 481 children per group allows for the detection of an effect size of 0.25 Standard Deviation (SD) for all other continuous outcomes, including linear growth, with 80% power and a maximum attrition rate of 15%. Assuming that the change in LAZ has a standard deviation of about 0.7, this equates to a difference of 0.175 *z*-score between any two groups.

The sample size calculation for the biochemistry subgroups was based on an expected standard deviation of serum zinc of no more than 3 μmol/L, 80% power, and a potential loss of 15% of samples due to attrition, sampling errors or insufficient blood collection. With these parameters, 58 children are required per group (i.e., 174 children total) to detect a difference of 1.68 μmol/L between any two sub-groups. With a sample size of 58 children per sub-group (i.e., 174 children total), we will have 80% power to detect a difference in EZP size of 0.67 mg/kg body weight, assuming a one-sided test, alpha of 0.05, standard deviation of 1.2, and potential loss of 15% of samples due to attrition or sampling errors. This effect size is consistent with findings from a study of zinc supplementation in Pakistani infants, which reported an effect size of 0.8 mg/kg body weight for EZP size [22].

### 2.10. Randomization and Double-Blinding

Children meeting the eligibility criteria and whose caregivers provided informed consent will be stratified by sex and then randomized into one of six groups using block randomization, in order to ensure even distribution of groups across time. Sealed opaque envelopes bearing the subject number and containing a paper with the group assignment and any subgroup assignments will be prepared by a person not involved in any study activities and codes will be stored in a secure computer file accessible only by two persons not involved in the project working at icddr,b.

At the time of allocation, the study personnel will open the envelope as per the specific child’s study identification numbers in a chronological way, and will record the specific code allocation in the infant’s clinical record forms and also in a register. Then, she/he will request the appropriate supplement from a person responsible for dispensing of supplements. All individuals involved in the trial (including parents, research staff and investigators) will be unaware of the intervention group assignment until the code is revealed when the data analysis is complete.

Given the distinct differences between powders and dispersible tablets, it will not be possible to blind study groups 1, 2, 3, and 6 from study groups 4 and 5. However, complete double-blinding will occur among study groups 1, 2, 3, and 6, and between study groups 4 and 5. Since participants in study group 3 will be required to consume different formulations on alternating days, and, because this characteristic distinguishes them from groups 1, 2, and 6, the powders for each of these groups will be packaged in two distinct colors. All participants in study groups 1, 2, 3, and 6 will be asked to consume the powders in different-colored sachets on alternating days. Neither the study personnel nor the participants will know the meaning of the different colors. Similarly, the dispersible zinc and placebo tablets will be manufactured with different codes marked directly on the tablets, and will be packaged in color-coded blister packs, so that participants in study group 5 consume tablets with a specific code/color for two weeks at baseline and 3 months, and tablets with a different code/color on all other days. Since this schedule distinguishes study group 5 from study group 4, all participants in study group 4 will also consume different marked/color-coded tablets for 2 weeks at baseline and 3 months; however, for this group, both types of tablets will contain 10 mg zinc.

### 2.11. Data Collection: Sociodemographics, Morbidity, and Anthropometry

At enrolment, trained study workers will collect data on background, socioeconomic status, household characteristics, assets, household composition, education, and food security using pretested questionnaires. The study workers will visit each study participant’s household twice weekly (i.e., Sunday/Wednesday or Monday/Thursday) to inquire about and record any morbidity that took place in the previous three to four days. It is necessary to visit the households twice weekly to document any diarrhea that occurred in the past 3 or 4 days and how it is being treated. If treatment was not initiated, the health worker will evaluate the health status of the child and make further recommendations as per the national strategy [17].

Diarrhea will be defined as three or more loose, liquid, or watery stools over a 24-hour period, separated in time from an earlier or subsequent episode by at least 2 consecutive diarrhea-free days [23,24]. Dysentery will be defined as any diarrheal episode in which the loose or watery stools contain visible blood [24,25]. Dehydration will be assessed using a standardized method established by the icddr,b [26]. Fever will be defined as an axillary temperature above 38.3 °C reported either by the caregiver or the study workers, who will be carrying thermometers. Acute upper respiratory infection will be defined as pharyngitis or rhinitis, both without rapid respiratory rate or chest in-drawing [27]. Acute lower respiratory infection will be defined as cough or difficulty breathing, rapid respiratory rate (>50 breaths/minute in infants 9–11 months of age and >40 breaths per minute among infants 12 months of age and older), and either a fever of >38.3 °C or chest retractions [27]. Hospitalization will be defined as an overnight stay in the hospital due to illness.

During the home visits, the study worker will inquire about the supplement consumption during the previous 3 or 4 days and continuous supplement consumption in the future will be encouraged. Leftover supplements (if any) along with the tablet strips/used sachets will be collected. The number consumed along with the number prescribed will be recorded.

Study anthropometrists who are trained in the anthropometry procedures will conduct all of the measurements at three central measurement sites at three individual field offices. Body weight and length of each child will be measured at enrolment and at 12 and 24 weeks. Body weight will be measured on a balance sensitive to 2 g (SECA, model No. 7281321009, Hamburg, Germany) and length will be measured to 0.1 cm using an infantometer (SECA, model No. 4161721009, Hamburg, Germany). The measurements will be performed in triplicate following standard procedures [28]. Workshops will be conducted every 6 months to standardize the methods used by the anthropometrist; workshops are also scheduled whenever any new anthropometrists are hired.

### 2.12. Data Collection: Biochemistry Sub-Group

The first 58 participants in all the six study groups will be enrolled in a biochemistry subgroup for measuring serum zinc, ferritin, sTfR, RBP, CRP, AGP and hemoglobin will be compared. At baseline and upon completion of the 24-week study period, a trained phlebotomist will collect 5 mL of venous whole blood from the antecubital vein using universal procedures and aseptic techniques. The tubes will be placed on ice immediately after drawing and sent to the local icddr,b laboratory for serum separation within 60 min for centrifugation at 3000 rpm for 10 min. Each serum sample will be aliquoted into two separate tubes (one for serum zinc, one for ferritin, sTfR, RBP, AGP, and CRP) and stored at −20 °C. The first set of frozen serum samples will later be shipped to Children’s Hospital Oakland Research Institute (CHORI) where serum zinc concentrations will be measured using Inductively Coupled Plasma Optical Emission Spectrometry (ICP-OES). The second set of frozen serum samples will be shipped to the VitMin Lab, Willstaett, Germany where ferritin, sTFR, RBP, AGP, and CRP will be measured by sandwich ELISA [29]. Hemoglobin concentration will be measured at the RFO using a Hemocue, HB 301 Analyzer (Hemocue AB, Angelholm, Sweden).

### 2.13. Data Collection: Exchangeable Zinc Pool (Ezp) Size Estimation

The EZP size will also be measured in the children in the biochemistry subgroup at baseline and at the end of the 24-week study period. The children will come to the iccddr,b’s Clinical Trial Unit (CTU) in Dhaka for this measurement. On the morning of the study day, a zinc isotopic tracer will be administered intravenously in the ante-cubital vein (~300 µg of ^67^Zn at baseline and ~400 µg ^70^Zn at endline). Half of the children will be randomized to receive one of these isotopes at baseline and the other one at the endline and vice versa. A 3-way stopcock will be attached to the end of the butterfly tubing and the zinc isotope solution will be infused over 2–3 min. To ensure that the entire isotope dose is infused, the syringe and tubing will be rinsed twice with equal volumes of normal saline. Precautions to avoid zinc contamination will be used for all of the biochemical procedures. The participants’ body weight at the time of isotope infusion at baseline and endline will also be recorded.

On the 3rd day after isotope infusion (day 4), ~20 mL urine samples will be collected at the homes of the participants by the mothers with assistance from the study personnel. Morning and evening samples will be collected for 4 consecutive days (days 4–7) for a total of 8 urine samples/participant. After thoroughly cleaning the child’s perineum, urine will be collected in zinc-free standard adhesive pediatric urine collection bags (Briggs Health Care, Des Moines, IA, USA and then transferred to zinc-free Nalgene bottles (Thermo Fisher Scientific, Waltham, MA, USA) and stored at −20 °C at the CTU of icddr,b. Urine samples, along with any dose losses, will be shipped to the University of Colorado Denver for analysis of isotope ratios and enrichment via Inductively Coupled Plasma-Mass-Spectrometry (ICP-MS).

Stable isotope enrichment data processing: the size of the EZP will be calculated by dividing the dose of the intravenous isotope (^67^Zn or ^70^Zn) infused by the enrichment value at the *y*-intercept of the linear regression of a semi-log plot of urine enrichment data from d 4–7 (4 days) [30,31]. EZP will be expressed both in absolute mass (mg) and relative to body weight (mg/kg).

## 3. Data Management and Analysis

### 3.1. Data Management

Study workers and their designated supervisor will review all pre-coded data collection forms on a daily basis before submitting the forms to the data entry team at icddr,b. All data will be entered by two or three Data Management Assistants and verified by the Data Management Officer. Any differences between entries, as identified by the database program, will be resolved by rechecking the original forms against both datasets. A common dataset will then be generated. The Data Management Officer will develop programs in Microsoft Access to check for inconsistent or implausible values of variables in the common dataset. Any aberrant values will be forwarded to the field office and resolved by checking the original data collection form or by a repeat home visit whenever possible. Outliers will be identified by visually inspecting box plots and/or histograms of individual continuous variables, and scatterplots of related variables. Outliers, which are clearly impossible or implausible values, will be corrected if possible, or recoded to missing if correction is not possible. Outliers, which are plausible or possible, will be maintained.

### 3.2. Data Analysis Principles

A participant flow diagram will be prepared in accordance with the 2010 CONSORT guidelines [32]. The primary analysis will be conducted on a ‘complete-case intention to treat’ basis (i.e., results for all children will be analysed according to the study group to which they were randomized regardless of any protocol violations). Data from participants who were lost to follow-up because of death, travel from the study site, or refusal to continue the trial will be included in the analysis if available. However, there will also be a secondary set of analyses, which will be restricted to the sub-set of participants who adhere well to the protocol (i.e., a “per protocol” analysis).

All statistical analyses will be two-sided, at 5% level of significance other than the variables related to EZP, which will be one-sided. Where more than 10% of observations are missing for a dependent variable, we will report the number of observations used in the analysis. We will also compare the baseline characteristics of participants who are lost to follow-up with those who remained in the trial for the duration of the follow-up period to identify any possible biases and include such characteristics as covariates in the statistical analysis if bias is detected.

With the exception of analyzing severe adverse events throughout the course of the trial, formal interim analyses that examine study progress for futility issues will not be conducted. If enrolment is slower than anticipated, the study/screening area will be expanded or other corrective measures will be taken.

All analyses will be performed using SAS version 9.4 (SAS Institute, Cary, NC, USA) or Stata version 14 (Stata Corp., College Station, TX, USA). The WHO 2006 Child Growth Standards will be used for age- and sex-standardization of child weight, length, arm circumference, and WLZ.

### 3.3. Monitoring of Data Collection

Previously established and validated standard operating procedures for data collection, monitoring and quality control measures will be used throughout the study [33]. Supervisors will conduct quality control checks by repeating 10% of the visits by the supervisors on the same day as the FRA for the morbidity surveillance as per the definitions as outlined in the manual of procedures. These visits will be carried out without informing the respective FRAs. For the anthropometry, if the measurements with the supervisors are found to be greater than 1 cm in length or 500 g in weight [26], then the measurements will be unacceptable and a newer episode of measurements will be conducted on the same day. If the same anthropometrist is found to make mistakes repeatedly, then the supervisor will ensure that a refresher training is completed by the anthropometrists. All weighing scales will be calibrated daily and infantometers weekly [26], and three measurements will be taken and the average value will be recorded. 

### 3.4. Data Analysis Methods

All variables measured at the time of enrolment (i.e., prior to the first intake of the study supplement) will be considered background or baseline characteristics. These variables include: infant sex, infant anthropometry, i.e., LAZ, WLZ, and weight-for-age, *z*-score (WAZ); proportion stunting, wasting and underweight), breastfeeding status, morbidity occurrence in the previous two weeks, hemoglobin concentration, concentrations of biochemical measures (i.e., ferritin, sTfR, CRP, AGP, RBP, serum zinc) among infants in the biochemistry subgroup, maternal age, maternal marital status, maternal education, maternal Body Mass Index (BMI), parity, household size and composition, household food security status, household characteristics needed to calculate a wealth index. These treatment-group baseline characteristics will be presented in a table. Frequencies and percentages will be reported for categorical data, and Pearson’s chi-square test of homogeneity will be used to compare differences between groups. The mean and standard deviation or the median and range will be reported for continuous variables, and differences will be compared using ANOVA or the Kruskal–Wallis test. If more than 10% of data are missing, the number of participants included in the analysis will be indicated. The following variables will be considered as effect modifiers: (1) LAZ at enrolment; (2) serum zinc concentration at enrolment; (3) iron status (i.e., ferritin corrected for inflammation and sTfR) at enrolment; (4) sex of infant; (5) household socioeconomic status, primarily wealth and maternal education; and (6) household food security status.

The incidence of diarrhea between groups will be compared using Poisson regression (SAS GENMOD procedure). Following the initial test of equality of all group means, pairwise comparisons between all groups will be conducted with the Tukey-Kramer test. Additionally, we will compare the difference in incidence of diarrhea among all participants in study groups 1–5 vs. group 6 using Scheffe’s test. The change in LAZ from enrolment to study completion will be compared between groups using analysis of covariance (ANCOVA, SAS General Linear Model (GLM) procedure). Following the initial test of equality of all group means, pairwise comparisons between all groups will be conducted with the Tukey–Kramer test. Additionally, we will compare the difference in incidence of diarrhea and changes in LAZ among all participants in study groups 1–5 vs. group 6 using Scheffe’s test. Baseline LAZ will be included as a covariate in these models.

Other secondary efficacy outcomes will be examined using the same approach. Logistic regression models (SAS LOGISTIC procedure) will be used to compare any dichotomous variables at specific time points. For repeated measures of continuous outcome variables, a linear mixed model (SAS MIXED or HPMIXED procedure) will be used to compare patterns in the variable across time among the groups. For categorical variables, a variant mixed model logistic regression (SAS GLIMMIX procedure) will be used.

All statistical models will include any baseline characteristics as covariates that were not equally distributed between study groups at enrolment or which can be assumed to be associated with the outcome. The effects of potential effect modifiers listed above will be assessed with an interaction term in the ANOVA/ANCOVA model. Interactions with a *p*-value < 0.05 will be further explored with stratified analyses.

## 4. Safety and Ethics

### 4.1. Data Safety Monitoring

A Data Safety Monitoring Board (DSMB) will be formed for safety monitoring throughout the trial. The DSMB will consist of three persons (a statistician, a Bangladeshi pediatrician, and an external expert in zinc nutrition of toddlers). The group will meet prior to the start of the trial to define severe adverse events (SAEs) and a procedure for reporting and analysis of SAEs. Preliminary SAEs include: deaths, hospitalizations (i.e., an overnight stay in the hospital because of illness), and severe acute reactions associated with consumption of the supplement. The DSMB will meet three times, at the beginning of the study, 6–9 months after the start of the trial, and a final meeting after all data are collected, cleaned and locked.

### 4.2. Discontinuation Procedures and Stopping Rules

Because MNPs and zinc supplements have both been proven to be safe and are widely used in Bangladesh (and elsewhere), participants will continue consuming their assigned regimen for the entire 24-week study period unless they no longer meet the inclusion criteria (e.g., a child develops severe acute malnutrition). We propose discontinuing any of the study arms if the number of severe adverse events and hospitalizations is significantly higher (*p* < 0.05) than any of the other study arms. However, the final set of stopping rules will be determined by the DSMB.

### 4.3. Ethical Approval

Ethical approval has been obtained from Children’s Hospital Oakland Research Institute’s Institutional Review Board (IRB) as well as the Research Review Committee and Ethical Review Committee that comprise icddr,b’s IRB. The IRB review process is underway at the University of Colorado Denver as well. The trial has been registered with clinicaltrials.gov and the trial registration number is NCT03406793. The protocol version is Version 1.04, 23 May 2017.

### 4.4. Confidentiality

Privacy, anonymity and confidentiality of data/information identifying the participants will be strictly maintained. All medical information, description of treatment, and results of the laboratory tests performed on the participants will be confidential. No one other than the investigators of this research will have access to the data. Data related to the study may be sent to collaborating institutions outside the country for analysis; however, any personal identifiable information will be withheld.

The parent(s) or legal guardians of the participants will freely be able to communicate with the Principal Investigator of this study (contact address will be provided in the consent form). Absolute confidentiality cannot be guaranteed, since research documents are not protected from subpoena. The Ethical Review Committee of icddr,b, which ensures the right of the study participants, reserves the right to prohibit access those information. The research sponsors (CHORI and the Bill and Melinda Gates Foundation) may also look at the research files and medical records. However, the participants’ name or any identity will not be disclosed in publications from this study.

### 4.5. Declaration of Interests

None of the authors have shown any financial and other competing interests for the overall trial and the study site.

### 4.6. Dissemination Policy

The data, results and other findings originating from this study will be published after approval by all investigators of the protocol. The International Committee of Medical Journal Editors guidelines will be used to establish authorship on papers.

## Figures and Tables

**Table 1:** Characteristics of study interventions.

Study Group	Description	Form	Micronutrient Content	Frequency of Supplementation
1	Standard Micronutrient Powder (MNP)	Powder	Vitamin A: 400 µg Vitamin D: 5 µg Vitamin E: 5 mg Vitamin C: 30 mg Thiamine: 0.5 mg Riboflavin: 0.5 mg Niacin: 6 mg Pyridoxine: 0.5 mg Vitamin B12: 0.9 mg Folate: 150 µg Iron: 10 mg Zinc: 4.1 mg Copper: 0.56 mg Selenium: 17.0 µg Iodine: 90 µg	Daily for 24 weeks
2	High zinc, low iron MNP	Powder	Same as study group 1, except with 10 mg zinc and 6 mg iron	Daily for 24 weeks
3	High zinc, low iron MNP; high-zinc, no-iron MNP on alternating days	Powder	Same as study group 1, except with 10 mg zinc, and 6 mg iron and no iron on alternating days	Daily for 24 weeks
4	Dispersible zinc supplement	Dispersible tablet	10 mg zinc	Daily for 24 weeks
5	Intermittent zinc supplement	Dispersible tablet	10 mg zinc	Daily for 14 days at baseline and 3 months, placebo tablet on all other days
6	Placebo powder	Powder	None	Daily for 24 weeks

## Project Status

As of 6 December 2017, study site preparation is ongoing.

## Appendix A. Consent Form

Protocol Title: A Randomized, Double-blind Community Trial of Supplementation of Varied Doses of Zinc in Micronutrient Powders in Young Bangladeshi Children.

Investigator’s name: Dr. Md. Munirul Islam

Organization: icddr,b

## Purpose of the Research

The purpose of this study is to determine the optimal physical form (e.g., powder or dispersible tablet), dose, and frequency of preventive zinc supplements for reducing diarrhea and improving growth in older infants and young children.

## Background

Zinc is a nutrient that is found in foods and is important for young children to grow and help to prevent diarrhea. We are conducting this study to learn more about the optimal physical form (e.g., powder or dispersible tablet), dose, and frequency of preventive zinc supplements and whether this amount of zinc will be adequate in reducing diarrhea and improving growth of your children. We also will like to know that how much zinc is stored in your children’s body. To allow your child to participate in this study, it will be necessary to give your written consent.

This is a research study. Research studies only include participants who choose to participate. As a parent/guardian of the study participant, you have the right to know about the procedures that will be used in this research study so that you can make the decision whether or not to participate. The information presented here is to make you better informed so that you may give or withhold your consent to participate in this research study. Please take your time to make your decision and discuss it with your family and friends.

## Why Invited to Participate in the Study?

You are being asked to allow your child to take part in this study because: (1) s/he is between the ages of 9–11 months; (2) s/he is adequately nourished, as indicated by the fact that his/her height and weight are within the normal range and (3) s/he does not have any chronic illness. We know that young children have the greatest benefit from zinc and also at the risk of deficiency in Bangladesh.

## Methods and Procedures

If you agree and allow your child to participate in the study, you expect the following procedures during the duration of the intervention, which will be 24 weeks as described below.

(i) Your child will be allocated to any one of the intervention groups as mentioned below by a method like lottery where none of us have any control over it:

1. Study group 1: Standard Micronutrient Powder (MNP) (15 micronutrients) will be supplemented daily. Your child will receive Red package on even days and Green package on odd days.
2. Study group 2: High Zinc low Iron MNP (10 mg zinc instead of 4.1 mg), and decreased iron (6 mg encapsulated iron instead of 10 mg) supplemented daily. Your child will receive Blue package on odd days and Yellow package on even days.
3. Study group 3: MNP with or without iron on alternating days. On alternating days, children will consume (Day 1) the High Zinc low Iron MNP described above (i.e., Group 2) followed by (Day 2) the same MNP composition but without iron. Your child will receive Orange package on odd days and Purple package on even days.
4. Study group 4: Daily zinc tablet: Dispersible tablet with 10 mg zinc only will be supplemented daily.
5. Study group 5: Intermittent zinc tablets. Dispersible tablet with 10 mg zinc daily for 2 weeks at the beginning and at the 3 month-point of the trial. Placebo dispersible tablets will be given on all other days to ensure study participants are blinded.
6. Study group 6: Placebo control group: Placebo powder will be supplemented daily. Your child will receive Brown package on odd days and White package on even days.

(ii) At the beginning of the study, we will ask you about your socioeconomic status, household characteristics, assets, household composition, education, and household food security. We will also measure your child’s height and body weight at this time, at 12 weeks and at 24 weeks or end of the study period.

(iii) We will visit your home twice a week throughout the study and will enquire about his/her wellbeing during preceding three days.

(iv) Every week, at one of these semi-weekly visits, we will provide the child’s caregiver with 7 days worth of the study regimen. If the child is in Study Groups 1, 2, 3, or 6, then we will ask you to feed the daily dose of MNP or placebo powder after mixing with the first few portions (preferably 1–2 tablespoons) of the food in a single meal 7 days a week. If the child is in Study Groups 4 or 5, then we will ask you to place the dispersible tablets in a half spoon of expressed breast milk or clean water, wait until the tablet dissolves, and then feed the whole content at once to the child.

(v) If during any household visits, we can see that your child is having diarrhea then we will provide you with therapeutic zinc and ORS and instruct to withhold the usual study regimen until the course of treatment has been completed (which is typically 10 days).

(vi) If your child experiences diarrhea (or any other illness) between the semi-weekly household visits, we will request you to bring the child to a day clinic at the field office where a doctor will examine your child and will treat with best care possible accordingly.

(vii) One 5 mL (equivalent to one tea-spoon) blood sample each will be collected from a vein in your child’s arm at the beginning and at the end of a 24-week intervention period to assess zinc, iron and vitamin A status and presence of infection. In addition, we will also measure the protein and fat contents, immunologic markers like a number of cytokines of your child’s body as the same time points.

(viii) Some children will be assigned to a sub-group where the total body zinc of the children will be measured. If your child may be allocated to this sub-group, then we will also measure the total zinc pool size or total body zinc contents by using zinc stable isotopes. For this part of the study, we will request you and your child to visit the Clinical Trial Unit of icddr,b and stay there for 6 h during the day time. During this time, zinc isotope tracer will be administered intravenously (~300 µg of ^67^Zn at baseline and ~400 µg ^68^Zn at endline). We will collect your child’s spot urine (each ~20 mL equivalent to 4 tea-spoon full) on this day during morning and evening and also on days 4, 5, 6 and 7 from your house during morning and evening on these days. This procedure will take place once at the beginning of the intervention period and the other one at the end of the intervention period.

## Risk and Benefits

Your child may have mild side effects while participating in this study. The Investigators may give you medicines to help lessen side effects. Many side effects go away soon after the study interventions or test solutions are stopped. There are no major risks associated with participation in this study. At the time of collection of the blood sample, your child will feel a momentary pain due to the needle prick. There is also a rare chance of bluish discoloration surrounding the prick site due to mild leakage of blood in the skin, and a very distant possibility of local or systemic infections or problems. However, we will take required precautions, including using of disposable syringes and needles, to prevent these problems. All blood samples will be obtained by a qualified health care professional. Five ml of blood (equivalent to one tea-spoonful) two times each will be obtained once from your child during the course of the study.

“Heavy” zinc or zinc isotopes are not radioactive and are non-toxic. There are no known risks associated with ingestion of “heavy” zinc. However, infusion of “heavy” zinc is momentarily uncomfortable and may result in bruising at the puncture site, but rarely causes infection.

You or your child may not benefit from taking part in this research if your child is allocated to Group No. 6. The information we get from this study will help us to understand that what could be the optimal physical form (e.g., powder or dispersible tablet), dose, and frequency of preventive zinc supplements for reducing diarrhea and improving growth in older infants and young children.

## Privacy, Anonymity and Confidentiality

We do hereby affirm that privacy, anonymity and confidentiality of data/information identifying you and your child will strictly be maintained. We will keep all medical information, description of treatment, and results of the laboratory tests performed on your child confidential. No one other than the investigators of this research will have access to the data. Data related to the study may be sent outside the country for analysis; however, any personal identifiable information will be held and processed under secured conditions, with access to limited appropriate staff of that organization.

You will be able to freely communicate with any investigator of this study (contact address to be provided). Absolute confidentiality cannot be guaranteed, since research documents are not protected from subpoena. Ethical Review Committee of icddr,b, who ensures the right of the study participants, reserves the right to access those information. The research sponsor (*Children's Hospital Oakland Research Institute and Bill and Melinda Gates Foundation*) may also look at your research files and medical record. Your child’s name or any identity will not be disclosed while publishing the results of this study.

## Future Use of Information

If there is any other future use of the data or biological samples for this study, we are asking for your permission now in doing so, and, if you agree, we affirm the privacy, anonymity and confidentiality of any data/information identifying your child. The blood and urine samples of your child will be shipped to Germany and USA for laboratory analysis. The samples will be preserved for five years there, if the blood and urine analysis is required to repeat.

## Right Not to Participate and Withdraw

Your participation in the study is voluntary, and you are the sole authority to decide for and against your child’s participation in this study. You will also be able to withdraw your child’s participation any time during the study. Refusal to take part in or withdrawal from the study will involve no penalty or loss of benefits or attention.

## Principle of Compensation

There are no financial benefits for participating in this study. However, you and your child shall be provided the best possible free treatment for any condition requiring treatment or any research related injuries.

## Answering Your Questions/Contact Persons

We will happily provide you further information about the study, if any, now or at a later time. You may communicate with the principal investigators of the study or her/his designated person at the contact address given below. If you have additional questions later, you may physically contact Dr. Md. Munirul Islam at the Dhaka Hospital of icddr,b (*Mohakhali Cholera Hospital*) or call him at the telephone No. +880-2-982-7001 to 10. Ext. 2352 or mobile No. +880-1-755-6286-24.

If you agree to our proposal of enrolling you/your child in our study, please indicate that by putting your signature or your left thumb impression at the specified space below

Thank you for your cooperation

_______________________________________   ____________________
Signature or left thumb impression of      Date
Parent/ Guardian/ Attendant
_______________________________________    ____________________
Signature or left thumb impression of the witness Date
_______________________________________   ___________________
Signature of the PI or his/her representative   Date
(NOTE: In case of representative of the PI, she/he shall put her/his full name and designation and then sign)
(Name and contact phone of IRB Secretariat, RA, M. A. Salam Khan, Phone No: +880-2-988-6498 or PABX: +880-2-982-7001-10 (ext. 3206)

## Appendix B. Study Timeline

		**Year 1**												**Year 2**												**Year 3**		
	Months	**1**	**2**	**3**	**4**	**5**	**6**	**7**	**8**	**9**	**10**	**11**	**12**	**13**	**14**	**15**	**16**	**17**	**18**	**19**	**20**	**21**	**22**	**23**	**24**	**25**	**26**	**27**
Study Activities																												
**Institutional Review Board IRB Approval**																												
**Staff Recruitment and Training**																												
**Study Preparation**																												
**Participant Enrollment**																												
**Study Procedure/Follow-Up**																												
**Laboratory Analysis**																												
**Sample Shipping**																												
**Data Entry and Analysis**																												
**Report Writing**																												
